# C-Reactive Protein-to-Platelet Inflammatory Index (CPII) and Symptom Severity Score for Early Differentiation of Odontogenic Cervicofacial Necrotizing Fasciitis from Odontogenic Abscesses: A Retrospective Cohort Study

**DOI:** 10.3390/dj14030162

**Published:** 2026-03-11

**Authors:** Marko Tarle, Igor Čvrljević, Koraljka Hat, Marina Raguž, Ivan Salarić, Ivica Lukšić

**Affiliations:** 1 Department of Maxillofacial and Oral Surgery, Dubrava University Hospital, 10000 Zagreb, Croatia; mtarle@kbd.hr (M.T.); icvrljevic@kbd.hr (I.Č.); khat@kbd.hr (K.H.); salaric@sfzg.unizg.hr (I.S.); 2 School of Dental Medicine, University of Zagreb, 10000 Zagreb, Croatia; 3 School of Medicine, University of Zagreb, 10000 Zagreb, Croatia; 4 Department of Neurosurgery, Dubrava University Hospital, 10000 Zagreb, Croatia; maraguz@kbd.hr; 5 School of Medicine, Catholic University of Croatia, 10000 Zagreb, Croatia

**Keywords:** necrotizing fasciitis, odontogenic abscess, cervicofacial infection, C-reactive protein, platelets, CPII

## Abstract

**Background/Objectives**: Early differentiation of odontogenic cervicofacial necrotizing fasciitis (NF) from odontogenic abscess (OA) is clinically challenging yet critical due to the need for urgent surgical and antimicrobial escalation. We evaluated whether a novel C-reactive protein-to-platelet inflammatory index (CPII = CRP/platelets), combined with a symptom-based Symptom Severity (SS) score, improves early discrimination of NF from OA. **Methods:** This retrospective cohort study included 234 hospitalized patients with cervicofacial odontogenic infections treated between January 2010 and December 2023 (25 NF, 209 OA). Admission clinical variables, SS and SIRS scores, and laboratory parameters were analyzed. CPII and established immunoinflammatory indices (including AISI, SII, NLR, PLR, and LMR) were calculated. Group comparisons were performed using nonparametric and categorical tests. Diagnostic performance was assessed by ROC analysis, and multivariable logistic regression evaluated independent associations with NF. **Results:** Compared with OA, NF patients were older (median 42 [IQR 35–59] vs. 35 [IQR 26–49] years; *p* = 0.0098) and more frequently had comorbidities (52% vs. 25.4%; OR 3.19; *p* = 0.0087). Trismus and dysphagia were more common in NF (84% vs. 60.8%, *p* = 0.0272; 88% vs. 53.6%, *p* = 0.0010), with higher SS and SIRS scores (both *p* < 0.0001). NF was associated with longer hospitalization (median 17 vs. 6 days; *p* < 0.0001) and more complications (40% vs. 5.7%; OR 10.94; *p* < 0.0001). CRP was markedly higher in NF (median 287 vs. 111.5 mg/L; *p* < 0.0001), platelets were lower (median 210 vs. 249 × 10^9^/L; *p* = 0.0091), and CPII was substantially higher (median 1.23 vs. 0.45; *p* < 0.0001). AISI did not differ between groups (*p* = 0.861). ROC analysis demonstrated excellent discrimination for SS score (AUC 0.9328, cut-off 12), CRP (AUC 0.9109, cut-off 221 mg/L), and CPII (AUC 0.9271, cut-off 0.75), whereas AISI showed limited discrimination (AUC 0.5108). In multivariable analysis, both SS score (adjusted OR 2.08 per 1 point) and CPII (adjusted OR 6.87 per 0.5 units) were independently associated with NF; the combined SS + CPII model achieved an AUC of 0.9726. **Conclusions:** CPII is a simple, admission-available biomarker that differentiates odontogenic cervicofacial NF from OA with excellent accuracy and provides strong complementary value when combined with SS score. AISI, despite prior utility for odontogenic abscess severity assessment, did not discriminate NF from OA in this cohort.

## 1. Introduction

Odontogenic infections remain among the most frequent causes of emergency presentation and hospital admission in oral and maxillofacial surgery. Large hospital-based and multicenter studies consistently report that more than 80–90% of cervicofacial infections are of odontogenic origin, most commonly arising from mandibular teeth [1,2,3,4]. While the majority of odontogenic infections are localized and respond to timely source control and antibiotic therapy, a clinically relevant subset progresses beyond the alveolar process and spreads through cervicofacial fascial planes and deep neck spaces, leading to airway compromise, sepsis, and life-threatening complications [1,3,5]. Contemporary inpatient cohorts demonstrate that approximately 40–45% of hospitalized patients with odontogenic cervicofacial infection meet the criteria for sepsis at presentation, highlighting the potential for rapid systemic deterioration even in infections of dental origin [4,6].

Cervicofacial necrotizing fasciitis (NF) represents the most catastrophic manifestation along this disease spectrum. NF is a rare but highly aggressive soft-tissue infection; although head and neck involvement accounts for only a small proportion of all cases, it carries disproportionate morbidity and mortality [7,8,9]. Reported mortality rates in cervical NF remain substantial, commonly ranging from 10% to over 30% despite modern surgical and intensive care support [8,9,10]. Outcomes are strongly influenced by host factors, particularly diabetes mellitus and other systemic comorbidities, as well as by the development of severe complications such as descending necrotizing mediastinitis (DNM), which is repeatedly identified as a pivotal determinant of prognosis [3,8,10]. Across multiple reviews and case series, odontogenic infection is consistently identified as the leading etiologic source of cervical NF, accounting for approximately 45–50% of reported cases [8,9,10].

Early recognition of cervicofacial NF remains challenging. Initial clinical manifestations frequently overlap with those of advanced odontogenic abscesses and deep neck infections, including swelling, trismus, dysphagia, fever, and laboratory evidence of systemic inflammation [4,9,11]. Classic cutaneous signs of necrosis are often absent in the early stage, and radiologic findings may be nonspecific or delayed relative to clinical progression [9,10]. As a result, misdiagnosis at initial presentation has been reported in up to 50–75% of cases, allowing rapid fascial necrosis to progress before definitive surgical management is initiated [9,11]. Although imaging plays an important role in anatomical assessment, reliance on radiologic confirmation alone may introduce clinically relevant delays when urgent surgical decision-making is required [10].

Laboratory-based diagnostic tools have therefore been explored as adjuncts to clinical assessment. The Laboratory Risk Indicator for Necrotizing Fasciitis (LRINEC) score was proposed to assist in early differentiation of NF from other soft-tissue infections [12]. However, subsequent studies focusing on head and neck disease have demonstrated variable diagnostic performance and limited sensitivity, supporting the view that LRINEC should not be used as a stand-alone tool in cervicofacial infections [9,10,11]. In parallel, increasing interest has been directed toward composite immunoinflammatory indices derived from routine complete blood count parameters, including the neutrophil-to-lymphocyte ratio (NLR), platelet-to-lymphocyte ratio (PLR), systemic immune-inflammation index (SII), and the Aggregate Index of Systemic Inflammation (AISI), as pragmatic markers of disease severity in odontogenic infections [13,14]. In hospitalized cohorts of odontogenic abscesses, AISI has demonstrated particularly strong prognostic value for abscess severity, outperforming CRP and supporting the concept that composite indices can capture clinically meaningful inflammatory phenotypes in odontogenic disease [14].

However, NF is biologically distinct from uncomplicated abscess formation. NF is characterized by profound systemic inflammation and dysregulated host response, and clinically this distinction is often reflected by markedly elevated CRP levels accompanied by relative thrombocytopenia [8,9,10,11]. This raises concerns regarding the applicability of platelet-integrating indices developed in abscess-dominant populations to the early identification of necrotizing soft-tissue infection, where platelet consumption and immune-coagulative interactions may alter index behavior.

To address this gap, we evaluated a simple, bedside-applicable biomarker, the C-reactive protein-to-platelet inflammatory index (CPII), which integrates the magnitude of the acute-phase response with a hematologic component that may reflect platelet consumption or dysregulated host response. We hypothesized that CPII, alone and in combination with symptom-based clinical stratification using the Symptom Severity (SS) score, would improve early differentiation of cervicofacial NF from OA. Accordingly, the aim of this retrospective cohort study was to compare clinical presentation and routinely available laboratory parameters between NF and OA, to evaluate the diagnostic performance of CPII relative to established inflammatory indices and CRP, and to assess the added discriminatory value of combining CPII with SS score.

## 2. Materials and Methods

### 2.1. Study Design, Setting, and Study Population

This retrospective cohort study was conducted at the Department of Maxillofacial and Oral Surgery, Dubrava University Hospital, Zagreb, Croatia. Consecutive hospitalized patients treated for cervicofacial odontogenic infections between January 2010 and December 2023 were retrospectively reviewed. The final study cohort comprised patients diagnosed with necrotizing fasciitis (NF) or odontogenic abscess (OA), as documented in the medical records by the treating surgical team. NF was defined as an odontogenic cervicofacial infection diagnosed by the treating surgical team and supported by surgical exploration/debridement with intraoperative findings consistent with necrotizing soft-tissue infection (fascial necrosis requiring debridement), as documented in operative and discharge records. OA was defined as an odontogenic cervicofacial infection managed as abscess (localized purulent collection clinically and/or radiologically) without an NF diagnosis and without intraoperative evidence of fascial necrosis. Odontogenic origin was established by clinical intraoral/dental examination and dental imaging (panoramic radiograph and/or CT when performed) identifying a dental focus contiguous with the cervicofacial infection. For severity-stratified analyses within the odontogenic abscess group, OA cases were further categorized as less severe odontogenic abscess (LSOA) or severe odontogenic abscess (SOA) according to the Symptom Severity (SS) score framework (LSOA: SS 0–8; SOA: SS 9–20), consistent with previously published applications of the SS score for odontogenic infection severity stratification (Table 1) [15]. The study protocol was approved by the Institutional Review Board of Dubrava University Hospital, Zagreb (approval no. 2022/1807-04, 28 July 2022). Informed consent was waived due to the retrospective design of the study and the use of anonymized, routinely collected clinical data.

### 2.2. Clinical Variables, Laboratory Parameters, and Index Definitions

Demographic and clinical variables extracted from records included gender, age, comorbidity status (present/absent), key symptom variables (trismus, dysphagia), anatomical localization of infection, length of hospitalization (days), and in-hospital complications (yes/no). Symptom burden and systemic involvement were quantified using the SS score and SIRS score as recorded at presentation (or derived from admission documentation where applicable). OA severity classification (LSOA vs. SOA) was defined a priori using SS score thresholds (0–8 vs. 9–20). Admission laboratory parameters were recorded from the first available blood test at hospital presentation, including white blood cell count, differential counts (neutrophils, lymphocytes, monocytes), platelet count, and C-reactive protein (CRP). Composite immunoinflammatory indices were calculated as follows: NLR (neutrophil-to-lymphocyte ratio) = neutrophils/lymphocytes; PLR (platelet-to-lymphocyte ratio) = platelets/lymphocytes; LMR (lymphocyte-to-monocyte ratio) = lymphocytes/monocytes; SII (systemic immune-inflammation index) = platelets × neutrophils/lymphocytes; AISI (Aggregate Index of Systemic Inflammation) = platelets × neutrophils × monocytes/lymphocytes; NLR+PLR = NLR + PLR. The proposed C-reactive protein-to-platelet inflammatory index (CPII) was defined as CPII = CRP (mg/L)/platelets (×10^9^/L). For graphical presentation, CRP, AISI, and CPII were plotted on a logarithmic scale to improve interpretability across their wide clinical ranges. Discharge decisions were made by the attending surgeon based on clinical improvement together with routine paraclinical monitoring. Patients were considered for discharge when they were hemodynamically stable and afebrile, demonstrated regression of local symptoms with adequate oral intake, had no signs of airway compromise or infection progression, and had no further indication for inpatient surgical procedures or intravenous antibiotics. Laboratory trends (white blood cell count and CRP) were reviewed to confirm improvement compared with admission. Imaging was not mandated as a discharge criterion and was repeated only when the clinical course suggested persistent or progressive infection.

### 2.3. Statistical Analysis

Continuous variables were assessed for distributional characteristics and are reported primarily as median (interquartile range, IQR); categorical variables are reported as counts and percentages. Between-group comparisons (NF vs. OA) were performed using Mann–Whitney U tests for continuous variables and χ^2^ tests or Fisher’s exact tests for categorical variables, as appropriate. For key dichotomous clinical variables, odds ratios (ORs) with 95% confidence intervals (CIs) were calculated. For severity-stratified analyses across LSOA, SOA, and NF, overall differences were evaluated using the Kruskal–Wallis test, followed by appropriate post hoc pairwise testing when overall significance was present. Diagnostic discrimination of candidate predictors (clinical scores and laboratory indices) for identifying NF vs. OA was assessed using receiver operating characteristic (ROC) analyses. The area under the ROC curve (AUC) was reported with 95% CIs, and the optimal cut-off for each predictor was determined using the Youden index, with corresponding sensitivity and specificity. To evaluate independent associations with NF, a prespecified multivariable logistic regression model was fitted including SS score and CPII as covariates. Effect sizes are reported as adjusted odds ratios with 95% CIs, with SS score modeled per 1-point increment and CPII modeled per clinically interpretable increments (0.5 units). Model discrimination was summarized using AUC derived from predicted probabilities. Missing data were handled using an available-case (complete-case per analysis) approach without imputation. Two-sided *p*-values < 0.05 were considered statistically significant.

## 3. Results

A total of 234 hospitalized patients with cervicofacial odontogenic infections were included. Necrotizing fasciitis (NF) was diagnosed in 25/234 patients (10.7%), whereas odontogenic abscess (OA) accounted for 209/234 patients (89.3%). Within the odontogenic abscess group, 96 patients were classified as less severe odontogenic abscess (LSOA) and 113 as severe odontogenic abscess (SOA) according to Symptom Severity score thresholds. For severity-stratified analyses, OA cases were subsequently analyzed alongside NF to assess clinical, laboratory, and outcome gradients across LSOA, SOA, and NF.

### 3.1. Clinical Characteristics and Outcomes

Compared with patients with OA, those with NF were generally older and had a higher prevalence of comorbidities. In the NF group, comorbidities included diabetes mellitus (*n* = 6), cardiovascular disease (*n* = 7) and active malignancy (oncologic patients; *n* = 3). NF cases presented more frequently with trismus and dysphagia, reflected in significantly higher SS and SIRS scores. Hospitalization was nearly three times longer, and complications were markedly more frequent among NF patients. Regarding abscess localization within the OA group, perimandibular (38.2%) and submandibular (26.5%) abscesses were observed most frequently, followed by pterygomandibular (12.3%) and buccal abscesses (10.2%); other localizations accounted for the remainder. Baseline demographic characteristics, clinical presentation, and outcomes are summarized in Table 2.

### 3.2. Laboratory Findings and Inflammatory Indices

NF was associated with higher white blood cell count (17.8 [14–21.5] vs. 13.4 [11–15.4] ×10^9^/L; *p* = 0.0002) and higher neutrophil count (14.6 [11.2–17.59] vs. 10 [8–12.6] ×10^9^/L; *p* = 0.0017), while lymphocyte count was lower (1.2 [0.6–1.52] vs. 1.5 [1.1–1.9] ×10^9^/L; *p* = 0.0073). CRP was markedly higher in NF than in OA (287 [231.6–365] vs. 111.5 [61.4–163.7] mg/L; *p* < 0.0001), and platelet counts were lower in NF (210 [149–264] vs. 249 [207–308] ×10^9^/L; *p* = 0.0091). The proposed C-reactive protein-to-platelet index (CPII) was significantly elevated in NF (1.23 [0.88–2.01] vs. 0.45 [0.22–0.65]; *p* < 0.0001). Among the composite indices, NLR was higher in NF (11.3 [5.11–20.12] vs. 7 [4.71–9.58]; *p* = 0.0097), whereas SII, PLR, LMR, and NLR+PLR did not differ significantly between groups (all *p* > 0.05). AISI did not differ between NF and OA (1305.37 [666.67–3385.48] vs. 1414.29 [840–2349.6]; *p* = 0.861) (Table 3).

### 3.3. Severity-Stratified Comparison (LSOA, SOA, and NF)

**Figure 1:**
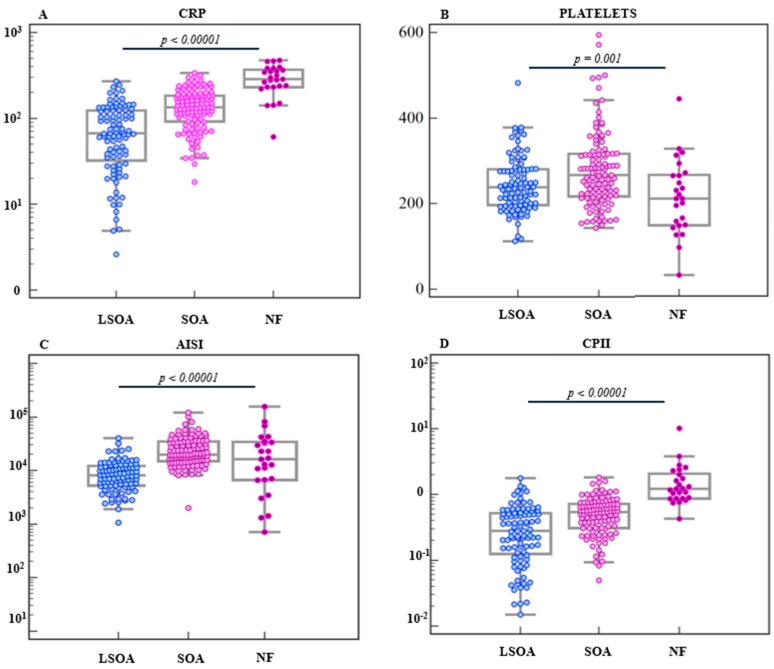
Inflammatory biomarkers across odontogenic abscess severity and necrotizing fasciitis. Individual patient-level values of (**A**) C-reactive protein (CRP), (**B**) platelet count, (**C**) Aggregate Index of Systemic Inflammation (AISI), and (**D**) C-reactive protein-to-platelet index (CPII) across LSOA, SOA, and NF. Horizontal bars indicate medians. CRP, AISI, and CPII are presented on a logarithmic scale.

In the severity-stratified analysis across LSOA, SOA, and NF, CRP increased stepwise (LSOA 66.75 [33.7–123.15], SOA 135 [92.2–183.5], NF 287 [231.6–365] mg/L; overall *p* < 0.0001), and CPII showed a similar gradient (LSOA 0.28 [0.13–0.52], SOA 0.54 [0.31–0.71], NF 1.23 [0.88–2.01]; overall *p* < 0.0001). Platelet counts differed across the three groups (LSOA 238 [197.5–279.5], SOA 267 [217–316], NF 210 [149–264] ×10^9^/L; overall *p* = 0.0011). AISI also differed overall across groups (LSOA 815.7 [527.68–1208.47], SOA 1978.26 [1493.4–3465], NF 1305.37 [666.67–3385.48]; overall *p* < 0.0001). Figure 1 visualizes individual patient-level values and highlights SOA vs. NF pairwise comparisons: CRP (*p* < 0.0001), platelets (*p* = 0.0022), AISI (*p* = 0.0673), and CPII (*p* < 0.0001).

### 3.4. Diagnostic Performance and Multivariable Analysis

**Figure 2:**
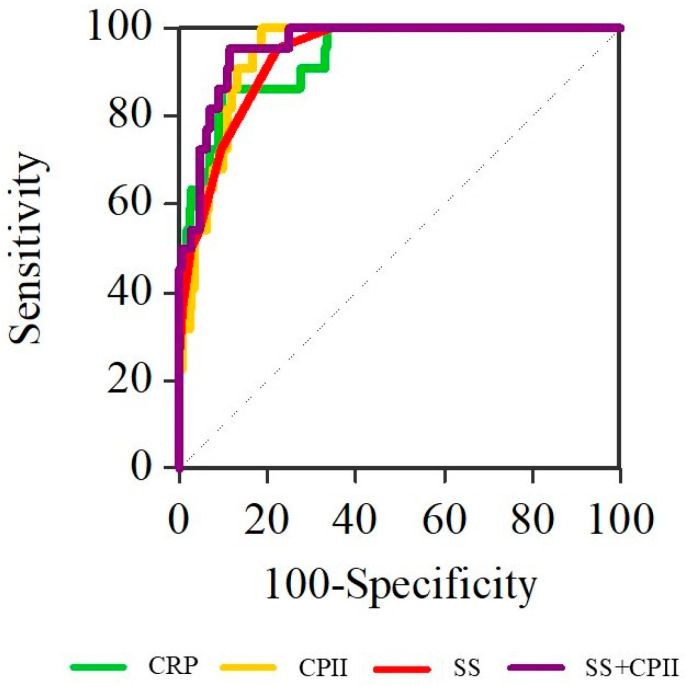
Receiver operating characteristic curves of CRP, CPII, SS score, and the combined SS score + CPII model for differentiation of necrotizing fasciitis from odontogenic abscess.

SS score, CRP, and CPII each demonstrated excellent discrimination of NF from OA. SS score yielded an AUC of 0.933 (95% CI 0.894–0.971) with an optimal cut-off of 12 (sensitivity 95.5%, specificity 77.5%). CRP yielded an AUC of 0.911 (95% CI 0.842–0.98) with an optimal cut-off of 221 mg/L (sensitivity 84%, specificity 90.4%). CPII achieved an AUC of 0.927 (95% CI 0.880–0.974) with an optimal cut-off of 0.75 (sensitivity 96%, specificity 81.3%). In contrast, AISI showed limited discrimination (AUC 0.511, 95% CI 0.364–0.658) (Table 4). In multivariable logistic regression including SS score and CPII (Table 5), both variables remained independently associated with NF: SS score (adjusted OR 2.08 per 1-point increase; 95% CI 1.44–3; *p* < 0.001) and CPII (adjusted OR 6.87 per 0.5-unit increase; 95% CI 2.63–17.93; *p* < 0.001). The combined SS score + CPII model showed very high discrimination (AUC 0.973, 95% CI 0.952–0.994) (Figure 2).

**Figure 3:**
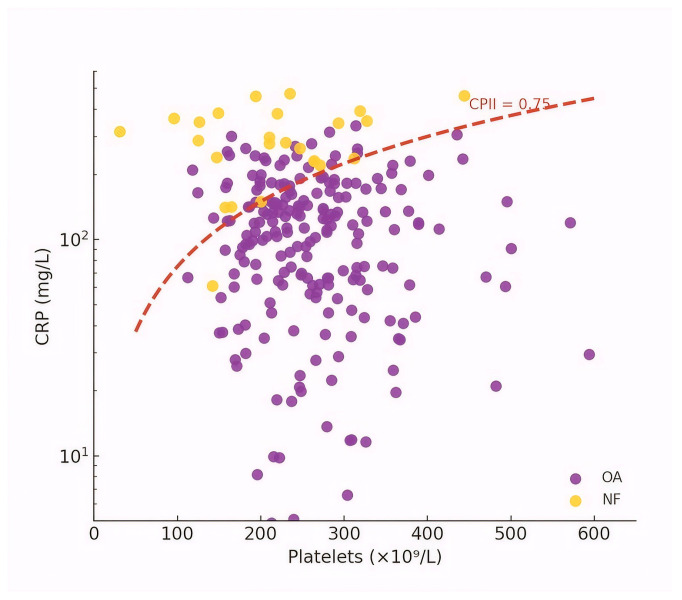
Relationship between C-reactive protein and platelet count with the CPII cut-off value for necrotizing fasciitis. The red dashed line represents the CPII cut-off value of 0.75 (CPII = CRP/platelets) derived from ROC analysis, indicating the threshold associated with a higher probability of necrotizing fasciitis.

The relationship between CRP and platelet count, together with the CPII cut-off derived from ROC analysis (0.75), is shown in Figure 3.

## 4. Discussion

Necrotizing fasciitis (NF) of the head and neck is an uncommon but highly aggressive soft-tissue infection associated with disproportionate morbidity and mortality [8,16,17]. Population-based data indicate that cervicofacial involvement is rare, accounting for approximately 1–10% of all NF cases, with an estimated incidence of around 2 cases per 1,000,000 inhabitants per year in Northern European populations [8,16]. Despite this low incidence, outcomes remain severe. Large contemporary syntheses report overall mortality rates ranging from 10% to over 30%, with pooled analyses of more than 1200 cervical NF cases demonstrating a mortality of approximately 13% and a mean hospital stay approaching one month, reflecting the resource-intensive nature of the disease [16].

Etiologically, odontogenic infections represent the single most common source of cervicofacial NF, accounting for approximately 45–50% of cases, followed by pharyngolaryngeal and tonsillar origins [8,16]. Odontogenic NF is of particular relevance to oral and maxillofacial practice because early clinical manifestations frequently resemble uncomplicated odontogenic abscesses, cellulitis, or deep neck space infections. As a result, misdiagnosis rates as high as 50–75% have been reported in early presentations, allowing rapid fascial necrosis to progress before definitive treatment is initiated [17,18,19,20,21]. Although overall mortality in odontogenic NF has been reported at approximately 10%, outcomes are strongly modified by host factors: patients with diabetes mellitus experience mortality rates exceeding 30%, underscoring the impact of systemic comorbidity even within this etiologic subgroup [8,16,17].

Severe complications critically determine prognosis. Descending necrotizing mediastinitis (DNM) is the most frequent and clinically decisive complication, reported in approximately one-quarter to one-third of cervicofacial necrotizing fasciitis cases and consistently associated with a two- to three-fold increase in mortality; notably, DNM occurred in 5 of 25 patients (20%) in our cohort, aligning closely with rates reported in large contemporary series [16,22]. Once mediastinal extension is accompanied by septic physiology, reported mortality rates escalate dramatically, reaching 40–60% in several series [23]. Major vascular complications, including internal jugular vein thrombosis and carotid sheath involvement, occur in approximately 5–8% of patients and further contribute to adverse outcomes [16,24]. Collectively, these data emphasize that cervicofacial NF, particularly of odontogenic origin, remains a low-incidence but high-impact disease, in which delayed recognition and progression beyond localized infection are the principal drivers of lethality.

Accordingly, the management paradigm for suspected NF is anchored in immediate action rather than diagnostic “watchful waiting.” Evidence supporting expedited surgery is unusually strong for a surgical infection: a 2020 systematic review and meta-analysis (6051 patients with necrotizing soft-tissue infections) demonstrated that mortality was significantly lower when surgery occurred within 6 h of presentation compared with delays beyond 6 h (19% vs. 32%; OR 0.43), and that surgery within 12 h also reduced mortality compared with later intervention (OR 0.41). Reviews further emphasize that delays in debridement are consistently associated with worse outcomes; for instance, one evidence-based management review summarizes data indicating that when surgery is delayed 24 h, mortality may increase nine-fold [25]. Parallel guidance documents converge on the same operational principle: early and aggressive surgical exploration/debridement coupled with broad-spectrum parenteral antibiotics should occur as soon as NF is suspected and should not be postponed while awaiting culture confirmation [20,26]. This “time is fascia” imperative is especially relevant in cervicofacial disease, where airway compromise and rapid spread along cervical fascial planes may necessitate early multidisciplinary coordination (maxillofacial surgery, ENT, anesthesia/ICU, infectious diseases, and occasionally thoracic surgery) [8,27].

Within this context, the clinical and laboratory phenotype observed in the present cohort aligns closely with the high-risk patterns described in the literature while also clarifying why an objective, rapidly available differentiation tool is needed. In our hospitalized cervicofacial odontogenic infection cohort, necrotizing fasciitis accounted for 10.7% of cases, reflecting the tertiary-care, hospital-based population rather than population incidence. Compared with odontogenic abscesses, NF was associated with a distinctly more severe initial clinical phenotype and a markedly worse short-term course. Patients with NF more frequently had systemic comorbidities (52% vs. 25.4%) and presented with advanced local symptoms, including trismus in 84% and dysphagia in 88% of cases, compared with 60.8% and 53.6%, respectively, among patients with OAs. Because trismus is a weighted component of the SS score (Table 1) and clinically often reflects masticator/deep-space involvement rather than the causative tooth alone, we retained trismus as a relevant functional marker and interpret it in conjunction with dysphagia and systemic markers rather than in isolation. Symptom-based severity was also substantially higher in NF, as reflected by elevated clinical scoring metrics at presentation. This higher initial disease burden translated into clinically meaningful outcome differences. Hospitalization for NF was nearly three-fold longer (median 17 vs. 6 days), and complications occurred in 40% of NF patients, compared with 5.7% in OAs. Collectively, these findings indicate that cervicofacial NF differs from OA not only in anatomical extent, but also in early clinical severity, systemic involvement, and short-term risk trajectory, reinforcing the importance of timely recognition and objective tools to support early differentiation. These findings are consistent with the established concept that cervicofacial NF is not simply a “more severe abscess,” but a distinct disease trajectory characterized by rapid systemic involvement and higher complication load, precisely the scenario in which early triage tools can change management timelines.

The present study further clarifies why reliance on single inflammatory indices may be misleading in necrotizing fasciitis and supports the mechanistic plausibility of CPII as a biomarker more closely aligned with the pathophysiology of NF. At admission, patients with NF demonstrated a pronounced systemic inflammatory response characterized by marked leukocytosis with neutrophilia and relative lymphopenia, consistent with an early hyperinflammatory and immunosuppressive shift. CRP showed a clear separation between NF and odontogenic abscesses, whereas platelet counts tended to be lower in NF, particularly when disease severity was taken into account, suggesting early consumption or inflammation-related platelet dysregulation. In contrast, patterns observed for AISI differed substantially. While AISI varied across the full spectrum of odontogenic infection severity, it did not reliably discriminate NF from OA, even in more advanced cases. This divergence highlights an important conceptual distinction: indices driven primarily by neutrophil dominance may reflect escalating local infection severity, whereas composite markers incorporating platelet behavior, such as CPII, appear more sensitive to the systemic, rapidly progressive inflammatory phenotype that characterizes NF. This “flattening” of AISI in NF is central to the present manuscript’s novelty because it highlights that a composite index that performs well for abscess severity can lose discrimination when thrombocytopenia becomes part of the biology. By design, AISI multiplies neutrophils, monocytes, and platelets divided by lymphocytes; thus, platelet reduction in NF can counterbalance the rise in neutrophils/monocytes and obscure the true severity signal.

This mechanistic divergence is particularly relevant in light of recent studies on odontogenic abscesses. In a recent investigation, AISI was identified as the strongest predictor of odontogenic abscess severity, outperforming CRP (AUC 0.9 vs. 0.74) with high sensitivity and specificity for severe abscesses, while a combined AISI + CRP approach further improved prediction of Systemic Inflammatory Response Syndrome (AUC 0.88) [14]. In contrast, findings from the present cohort indicate that when the disease process shifts from severe odontogenic abscess to necrotizing soft-tissue infection, the platelet component becomes pathophysiologically informative in the opposite direction. Rather than contributing to higher AISI values with increasing abscess severity, platelet depletion in necrotizing fasciitis may act as a “severity penalty” within AISI, thereby degrading its discriminatory performance (AUC 0.511 in the NF vs. OA ROC analysis).

This interpretation is well supported by contemporary platelet biology in severe infection. Platelets are now recognized as key immune and hemostatic mediators in sepsis, participating in immunothrombosis, endothelial interactions, and microvascular thrombus formation; in parallel, thrombocytopenia is common in severe systemic infection and can reflect consumption, disseminated intravascular coagulation, immune-mediated destruction, and marrow suppression [28,29,30]. Importantly, thrombocytopenia has also been linked to adverse outcomes in NF specifically; for example, a large retrospective analysis identified subsequent thrombocytopenia as associated with higher mortality in NF [31,32]. Against this biological background, the observed fall (or failure to rise) in platelets in NF in our dataset is not merely a statistical curiosity; it is a clinically meaningful feature that can be leveraged diagnostically.

The CPII concept operationalizes the clinically observed pattern of disproportionate CRP elevation accompanied by relative platelet decline into a single, bedside-computable index that appears to align more closely with the pathophysiology of necrotizing fasciitis than platelet-containing multiplication indices. CPII was substantially higher in NF than OA (median 1.23 vs. 0.45; *p* < 0.0001), and it increased stepwise across LSOA, SOA, and NF (0.28 → 0.54 → 1.23; overall *p* < 0.0001), mirroring the expected escalation from localized abscess to necrotizing infection. In ROC analyses CPII demonstrated excellent discrimination of NF from OA (AUC 0.927), comparable to CRP alone (AUC 0.911).

Importantly, CPII retained a strong and independent association with NF when evaluated alongside symptom-based severity measures in multivariable analyses, indicating that it provides information beyond clinical scoring alone. When combined with the SS score, CPII contributed to a model with excellent overall discriminatory capacity, underscoring the complementary value of integrating objective laboratory markers with clinical assessment. Figure 3 provides an intuitive visualization of this behavior by mapping CRP against platelet count and showing how the CPII threshold partitions the dataset: NF cases cluster in the high-CRP/low-platelet region consistent with a hyperinflammatory, consumptive phenotype. Taken together, these findings support CPII as a biologically plausible and empirically robust “bridge marker” linking systemic inflammatory intensity (CRP) with a key host-response component (platelet consumption/dysregulation) that is under-captured by existing odontogenic abscess severity indices.

From a clinical implementation perspective, the combined clinical–laboratory strategy suggested by these findings has broad applicability, as it relies on parameters that are routinely available in most emergency and acute care settings. SS scoring captures key functional red flags, such as trismus and dysphagia, which reflect deep-space involvement and potential airway compromise, while standard laboratory markers can be obtained concurrently without delaying triage or early management decisions. This pragmatic integration supports use across diverse institutional settings, including centers without immediate access to advanced imaging or subspecialty consultation.

CPII can be calculated immediately once CRP and platelet count are available, without requiring specialized assays. The high discrimination of SS score and CPII together implies a pragmatic triage approach: patients presenting with cervicofacial odontogenic infection and an SS score ≥ 12 and/or CPII ≥ 0.75 constitute a subgroup with substantially higher probability of NF in this dataset and could be prioritized for urgent cross-sectional imaging, early senior surgical review, and rapid escalation to operative exploration when clinical concern persists.

This is consistent with broader NF guidance that cautions against overreliance on laboratory scoring tools that may delay definitive management, especially in the head and neck where some commonly used indices (e.g., LRINEC) have limited sensitivity and should be considered adjunctive rather than exclusionary [11,21,33]. In other words, CPII should be framed not as a replacement for clinical judgment or imaging, but as a rapid, objective “risk amplifier” that supports the low threshold for early intervention demanded by NF’s time-dependent mortality profile.

Several limitations should be recognized when positioning CPII for translation into practice. The study is retrospective and single-center with a relatively small NF sample, which limits model stability and may inflate performance estimates, particularly because cut-offs/AUCs were derived and tested in the same cohort and internal resampling validation (e.g., bootstrapping) was not performed; external validation in independent cohorts is therefore essential before CPII cut-offs are treated as universal. Additionally, while CPII performed strongly for differentiation, the present analysis does not yet establish whether CPII is primarily a diagnostic marker of NF, a prognostic marker of adverse outcomes within NF, or both; future work could evaluate CPII against clinically meaningful endpoints such as ICU admission, mediastinal extension, number of debridements, or mortality. Finally, because platelet counts may be influenced by comorbidities, medications (e.g., chemotherapy or other marrow-suppressive therapies, chronic liver disease or hematologic disorders, and pre-existing thrombocytopenia), and timing relative to symptom onset, prospective assessment of CPII trajectories (serial values) could clarify whether rising CPII precedes overt necrotizing signs, which would further strengthen its early warning utility. Nonetheless, in a clinical domain where delayed recognition can be catastrophic and where even modern series still report double-digit mortality and high complication rates, a simple index that combines CRP intensity with platelet depletion and that demonstrably complements a symptom-based severity score represents a clinically coherent and potentially high-impact contribution.

## 5. Conclusions

This study identifies CPII as a strong and readily accessible laboratory marker for early differentiation of cervicofacial odontogenic NF from OA, performing comparably to CRP and providing excellent discrimination when combined with the SS score. In contrast, AISI, despite prior usefulness in predicting OA severity, did not discriminate NF from OA in this cohort. These findings support CPII as a clinically pragmatic adjunct to symptom-based assessment for early recognition of necrotizing disease.

## Figures and Tables

**Table 1:** The Symptom Severity (SS) score for odontogenic infections, as developed by Sainuddin et al. [15].

Criteria		Score	Max Score
Systemic Inflammatory Response Syndrome (SIRS)	Temperature > 38.3 °C	1	4
	Heart rate > 90 bpm	1	
	RR > 20/min	1	
	WBC < 4 or > 12 × 10^9^/L	1	
Trismus	Moderate < 2 cm	3	4
	Severe < 1 cm	4	
Dysphagia	Mild—able to swallow most foods	2	5
	Moderate—unable to swallow fluids	4	
	Severe—drooling saliva	5	
Collection in 1 fascial space	Low severity (canine, vestibular)	1	5
	Moderate severity (buccal)	2	
	High severity (all other spaces)	4	
Collection in 2 or more fascial spaces		5	
Sign of dehydration (BP/Urea/Skin turgor)		1	2
Comorbidities: diabetes mellitus, immunocompromised status, known or suspected chronic alcohol misuse		1	
Total Score			20

SIRS, Systemic Inflammatory Response Syndrome; BP, blood pressure; RR, respiratory rate; WBC, white blood cells.

**Table 2:** Baseline demographic characteristics, clinical presentation, and outcomes of patients with necrotizing fasciitis and odontogenic abscess.

Characteristic	NF	OA	OR (95% CI)	*p*
Age, years	42 (35–59)	35 (26–49)	—	0.0098
Male sex, *n* (%)	19/25 (76)	118/209 (56.5)	2.44 (0.94–6.36)	0.0845
Comorbidities present, *n* (%)	13/25 (52)	53/209 (25.4)	3.19 (1.37–7.42)	0.0087
Trismus present, *n* (%)	21/25 (84)	127/209 (60.8)	3.39 (1.12–10.23)	0.0272
Dysphagia present, *n* (%)	22/25 (88)	112/209 (53.6)	6.35 (1.84–21.87)	0.0010
SS score	14.5 (12.25–16.75)	9 (6–11)	—	<0.0001
SIRS score	3 (2–3.75)	1 (0–2)	—	<0.0001
Hospital stay, days	17 (10–23)	6 (4–7)	—	<0.0001
Complications, *n* (%)	10/25 (40)	12/209 (5.7)	10.94 (4.07–29.45)	<0.0001

NF, necrotizing fasciitis; OA, odontogenic abscess; SS, Symptom Severity score; SIRS, Systemic Inflammatory Response Syndrome.

**Table 3:** Admission laboratory parameters and immunoinflammatory indices in necrotizing fasciitis and odontogenic abscess.

Laboratory Parameter	Necrotizing Fasciitis (NF)	Odontogenic Abscess (OA)	*p*
White blood cell count, ×10^9^/L	17.8 (14–21.5)	13.4 (11–15.4)	0.0002
Neutrophils, ×10^9^/L	14.6 (11.2–17.59)	10 (8–12.6)	0.0017
Lymphocytes, ×10^9^/L	1.2 (0.6–1.52)	1.50 (1.1–1.9)	0.0073
Monocytes, ×10^9^/L	0.72 (0.4–1)	0.87 (0.6–1.1)	0.0855
Platelets, ×10^9^/L	210 (149–264)	249 (207–308)	0.0091
CRP, mg/L	287 (231,6–365)	111.5 (61.4–163.7)	<0.0001
CPII	1.23 (0.88–2.01)	0.45 (0.22–0.65)	<0.0001
SII	2552 (1120–5138.13)	1671.33 (1171.5–2592.86)	0.2204
NLR	11.3 (5.11–20.12)	7 (4.71–9.58)	0.0097
PLR	210 (110–292.11)	175.38 (128.33–231.25)	0.4896
AISI	1305.37 (666.67–3385.48)	1414.29 (840–2349.6)	0.8610
LMR	1.5 (1.11–2.4)	1.78 (1.25–2.6)	0.5297
NLR + PLR	225.7 (114.14–303.68)	180.15 (133.76–240.36)	0.4550

CRP, C-reactive protein; CPII, C-reactive protein-to-platelet inflammatory index; NLR, neutrophil-to-lymphocyte ratio; PLR, platelet-to-lymphocyte ratio; LMR, lymphocyte-to-monocyte ratio; SII, systemic immune-inflammation index; AISI, Aggregate Index of Systemic Inflammation.

**Table 4:** Diagnostic performance of clinical and laboratory parameters for differentiation of necrotizing fasciitis from odontogenic abscess (ROC analysis).

Predictor	*n*	AUC (95% CI)	Cut-off	Sensitivity (%)	Specificity (%)
SS score	231	0.933 (0.894–0.971)	12	95.5	77.5
CRP	234	0.911 (0.842–0.98)	221	84	90.4
CPII	234	0.927 (0.88–0.974)	0.75	96	81.3
SIRS	231	0.841 (0.772–0.911)	2	95.5	55
NLR	234	0.658 (0.518–0.798)	9.667	64	76.1
AISI	234	0.511 (0.364–0.658)	2967.3	36	82.3

SS, Symptom Severity score; CRP, C-reactive protein; CPII, C-reactive protein-to-platelet inflammatory index; SIRS, Systemic Inflammatory Response Syndrome; NLR, neutrophil-to-lymphocyte ratio; AISI, Aggregate Index of Systemic Inflammation.

**Table 5:** Multivariable logistic regression analysis for predictors of necrotizing fasciitis.

Predictor	Adjusted OR	95% CI	*p*
SS score (per 1 point)	2.08	1.44–3.00	<0.001
CPII (per 0.5 units)	6.87	2.63–17.93	<0.001

SS, Symptom Severity score; CPII, C-reactive protein-to-platelet inflammatory index.

## Data Availability

All data generated or analyzed during this study are included in the published article.

## Abbreviations

The following abbreviations are used in this manuscript:

AISI	Aggregate Index of Systemic Inflammation
CRP	C-Reactive Protein
CPII	C-Reactive Protein-to-Platelet Inflammatory Index
ICU	Intensive Care Unit
LMR	Lymphocyte-to-Monocyte Ratio
LSOA	Less Severe Odontogenic Abscess
NF	Necrotizing Fasciitis
NLR	Neutrophil-to-Lymphocyte Ratio
OA	Odontogenic Abscess
PLR	Platelet-to-Lymphocyte Ratio
SII	Systemic Immune-Inflammation Index
SIRS	Systemic Inflammatory Response Syndrome
SOA	Severe Odontogenic Abscess
SS	Symptom Severity Score
